# Supplementary material for: HIVprotI: an integrated web based platform for prediction and design of HIV proteins inhibitors
Source: J Cheminform. 2018 Mar 9;10:12. doi: 10.1186/s13321-018-0266-y (PMC5845081; doi:10.1186/s13321-018-0266-y)
Supplement: Supplementary file 2 — Additional file 2. Source code of HIVProtI web server. [file 13321_2018_266_MOESM2_ESM.zip › HIVprotI_Source-code/hivprotI/weka-3-6-6/doc/weka/gui/beans/KnowledgeFlow.html]

KnowledgeFlow


---


|  |  |  |
| --- | --- | --- |
| **Overview** \| **Package** \| **Class** \| **Tree** \| **Deprecated** \| **Index** \| **Help** \| **Weka's home** | |  |
| **PREV CLASS**   **NEXT CLASS** | **FRAMES**    **NO FRAMES**     **All Classes** |  |
| SUMMARY: NESTED \| FIELD \| CONSTR \| METHOD | DETAIL: FIELD \| CONSTR \| METHOD |  |


---


## weka.gui.beans Class KnowledgeFlow

```
java.lang.Object
  weka.gui.beans.KnowledgeFlow
```

---

``` public class KnowledgeFlow extends java.lang.Object ```

Startup class for the KnowledgeFlow. Displays a splash screen.

**Version:**
:   $Revision: 7059 $

**Author:**
:   Mark Hall

---

**Constructor Summary**

`KnowledgeFlow()`


| **Method Summary** | |
| --- | --- |
| `static void` | `main(java.lang.String[] args)`             Shows the splash screen, launches the application and then disposes the splash screen. |
| `static void` | `startApp()`             Static method that can be called from a running program to launch the KnowledgeFlow |

**Methods inherited from class java.lang.Object**

`equals, getClass, hashCode, notify, notifyAll, toString, wait, wait, wait`

**Constructor Detail**

### KnowledgeFlow

```
public KnowledgeFlow()
```


**Method Detail**

### startApp

```
public static void startApp()
```

:   Static method that can be called from a running program
    to launch the KnowledgeFlow

---


### main

```
public static void main(java.lang.String[] args)
```

:   Shows the splash screen, launches the application and then disposes
    the splash screen.

    :   **Parameters:**: `args` - the command line arguments


---


|  |  |  |
| --- | --- | --- |
| **Overview** \| **Package** \| **Class** \| **Tree** \| **Deprecated** \| **Index** \| **Help** \| **Weka's home** | |  |
| **PREV CLASS**   **NEXT CLASS** | **FRAMES**    **NO FRAMES**     **All Classes** |  |
| SUMMARY: NESTED \| FIELD \| CONSTR \| METHOD | DETAIL: FIELD \| CONSTR \| METHOD |  |


---
